# Artificial Intelligence for the Estimation of Visual Acuity Using Multi-Source Anterior Segment Optical Coherence Tomographic Images in Senile Cataract

**DOI:** 10.3389/fmed.2022.871382

**Published:** 2022-05-17

**Authors:** Hyunmin Ahn, Ikhyun Jun, Kyoung Yul Seo, Eung Kweon Kim, Tae-im Kim

**Affiliations:** ^1^Department of Ophthalmology, Institute of Vision Research, Yonsei University College of Medicine, Seoul, South Korea; ^2^Corneal Dystrophy Research Institute, Yonsei University College of Medicine, Seoul, South Korea; ^3^Saevit Eye Hospital, Goyang, South Korea

**Keywords:** artificial intelligence, cataract, convolutional neural network, optical coherence tomography, visual acuity

## Abstract

**Purpose:**

To investigate an artificial intelligence (AI) model performance using multi-source anterior segment optical coherence tomographic (OCT) images in estimating the preoperative best-corrected visual acuity (BCVA) in patients with senile cataract.

**Design:**

Retrospective, cross-instrument validation study.

**Subjects:**

A total of 2,332 anterior segment images obtained using swept-source OCT, optical biometry for intraocular lens calculation, and a femtosecond laser platform in patients with senile cataract and postoperative BCVA ≥ 0.0 logMAR were included in the training/validation dataset. A total of 1,002 images obtained using optical biometry and another femtosecond laser platform in patients who underwent cataract surgery in 2021 were used for the test dataset.

**Methods:**

AI modeling was based on an ensemble model of Inception-v4 and ResNet. The BCVA training/validation dataset was used for model training. The model performance was evaluated using the test dataset. Analysis of absolute error (AE) was performed by comparing the difference between true preoperative BCVA and estimated preoperative BCVA, as ≥0.1 logMAR (AE_≥0.1_) or <0.1 logMAR (AE_ <0.1_). AE_≥0.1_ was classified into underestimation and overestimation groups based on the logMAR scale.

**Outcome Measurements:**

Mean absolute error (MAE), root mean square error (RMSE), mean percentage error (MPE), and correlation coefficient between true preoperative BCVA and estimated preoperative BCVA.

**Results:**

The test dataset MAE, RMSE, and MPE were 0.050 ± 0.130 logMAR, 0.140 ± 0.134 logMAR, and 1.3 ± 13.9%, respectively. The correlation coefficient was 0.969 (*p* < 0.001). The percentage of cases with AE_≥0.1_ was 8.4%. The incidence of postoperative BCVA > 0.1 was 21.4% in the AE_≥0.1_ group, of which 88.9% were in the underestimation group. The incidence of vision-impairing disease in the underestimation group was 95.7%. Preoperative corneal astigmatism and lens thickness were higher, and nucleus cataract was more severe (*p* < 0.001, 0.007, and 0.024, respectively) in AE_≥0.1_ than that in AE_ <0.1_. The longer the axial length and the more severe the cortical/posterior subcapsular opacity, the better the estimated BCVA than the true BCVA.

**Conclusions:**

The AI model achieved high-level visual acuity estimation in patients with senile cataract. This quantification method encompassed both visual acuity and cataract severity of OCT image, which are the main indications for cataract surgery, showing the potential to objectively evaluate cataract severity.

## Introduction

Cataract is the leading cause of blindness, with ~12.6 million cases of cataract worldwide (1). The visual impairment caused by cataract can be treated with advanced cataract surgery, which can ensure a progressively better quality of vision and fewer complications than in the past (2–5). The most important indications for cataract surgery are preoperative visual acuity and cataract grading, and advances in surgical technology have expanded the scope of the surgery to even include less severe cataracts (5).

Although the cataract grading system shows a good correlation with surgical difficulty, indicating that the surgery becomes more challenging as the cataract grading increases (6, 7), it shows limitations in reflecting the patient's visual symptoms, especially in cases with nuclear cataract and cortical opacity (8). Cataract grading depends on the subjective competence of the investigator (9). However, visual acuity reflects the patient's symptoms and influences surgical difficulty; therefore, the surgery becomes more challenging also as the visual acuity decreases (10, 11). Moreover, preoperative visual acuity can serve as an important predictor of postoperative vision in various diseases (12–14).

Artificial intelligence (AI) is being increasingly used in medicine, and ophthalmology is one of the most active fields for its clinical application (15). Recent studies have attempted to use AI for cataract grading with various methods, including slit-lamp photography, fundus photography, and optical coherence tomography (OCT) (16–22), and the results suggest that AI-based cataract grading shows acceptable performance with 70–90% accuracy. However, an AI-based approach for evaluation of visual acuity in patients with cataracts is still lacking. An approach linking objective image data with the subjective symptoms represented by visual acuity is particularly relevant, since the resultant method would encompass both visual acuity and cataract grade, which are the main indications for cataract surgery. Therefore, we attempted to implement an AI model that can evaluate cataract severity based on visual acuity by using multi-source OCT data and to assess the applicability of this AI model in actual clinical practice.

## Methods

The study was conducted at the Department of Ophthalmology, Severance Hospital, Yonsei University College of Medicine in accordance with the ethical standards of the Declaration of Helsinki, and institutional review board approval was obtained for the study protocol (4-2021-1697). The institutional review boards waived the need for informed consent because of the retrospective and de-identified nature of the study.

### Participants and Dataset

**Figure 1:**
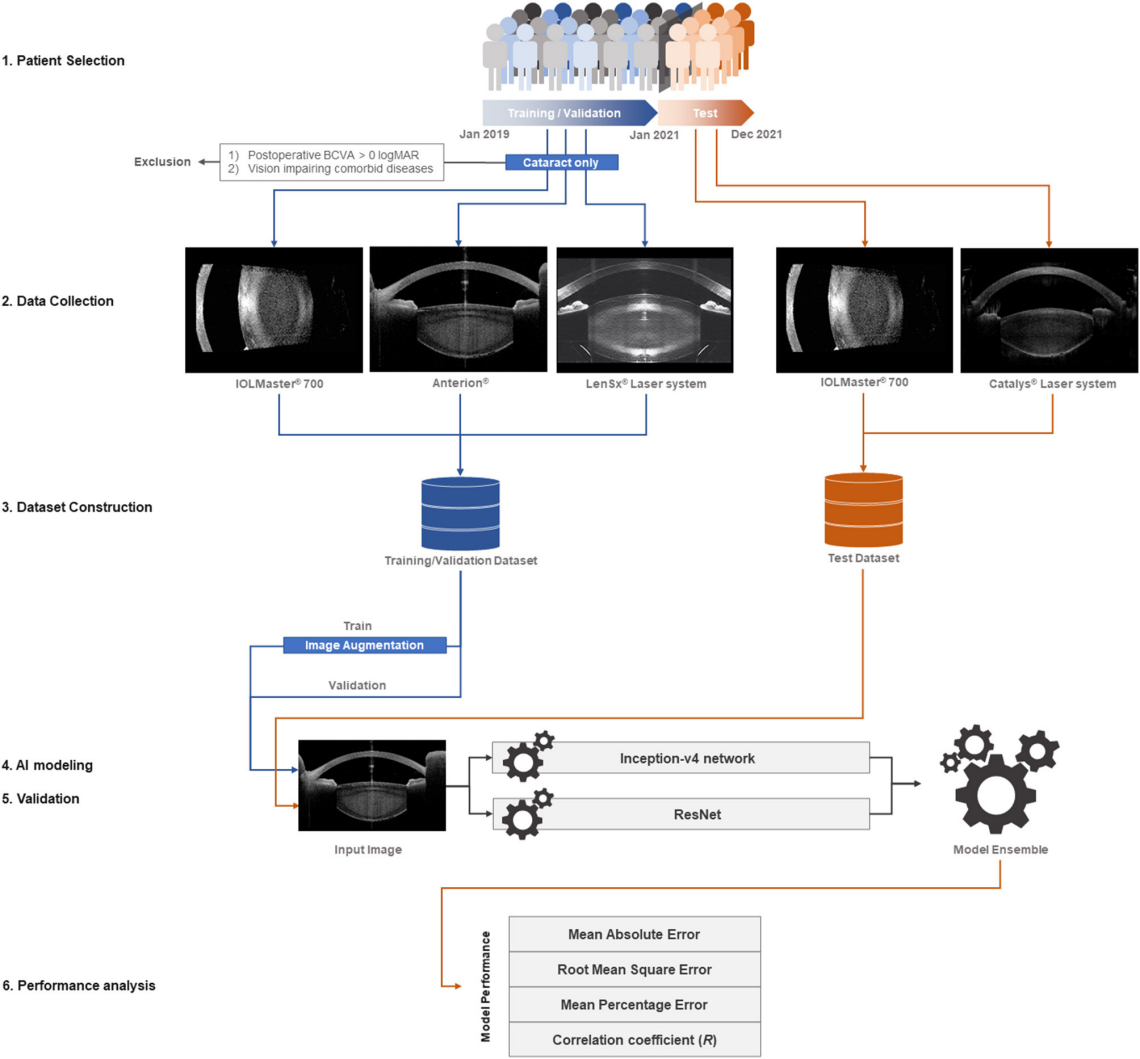
Study flow diagram. For the training/validation dataset, we collected 2,332 anterior segment OCT images of 2,332 eyes in as many patients with isolated senile cataract, whose 1-month postoperative best-corrected visual acuity (BCVA) was 0.0 logMAR or better. The images were obtained between January 2019 and December 2020 using swept-source OCT (ANTERION®), optical biometry for intraocular lens calculation (IOLMaster® 700), and a femtosecond laser platform (LenSx® Laser System). With this process, we aimed to develop a pure cataract analyzer. For the test dataset, we collected 1,002 anterior segment OCT images of 1,002 eyes in as many patients who underwent cataract surgery between January 2021 and December 2021 to evaluate the application of the analyzer in actual clinical practice. The images were obtained using optical biometry (IOLMaster® 700) and a different femtosecond laser platform (CATALYS™ Precision Laser System). The multi-source OCT images varied in size, contour, and direction; an image augmentation method (ImageDataGenerator from the Keras library) was used for the training dataset. The AI modeling was based on an ensemble of the Inception-v4 convolutional neural network (CNN) and ResNet via the stacking technique. The mean absolute error, root mean square error, mean percentage error, and correlation coefficient between the actual and predicted preoperative BCVA were calculated to evaluate the model performance.

All medical records of patients who underwent cataract surgery between January 2019 and December 2021 were reviewed. The demographic and clinical information of the patients, including age, sex, and clinical history, was collected. We defined distinct inclusion criteria for the training/validation and test datasets. For the training/validation dataset, we collected 2,332 anterior segment OCT images of 2,332 eyes in 2,332 patients with senile cataract alone whose 1-month postoperative best-corrected visual acuity (BCVA) was 0.0 logMAR or better; the images were obtained between January 2019 and December 2020 by using swept-source OCT (ANTERION® swept-source OCT; Heidelberg Engineering, Heidelberg, Germany), optical biometry for intraocular lens calculation (IOLMaster® 700; Carl Zeiss Meditec AG, Jena, Germany), and a femtosecond laser platform (LenSx® laser system; Alcon Laboratories, Inc., Fort Worth, TX, USA). Through this process, we aimed to develop a pure cataract analyzer. In the test dataset, to evaluate the application of the analyzer in actual clinical practice, we collected 1,002 anterior segment OCT images of 1,002 eyes of 1,002 patients who underwent cataract surgery between January 2021 and December 2021; the images were obtained using optical biometry (IOLMaster® 700) and another femtosecond laser platform (CATALYS™ Precision Laser System; Johnson & Johnson Inc., New Brunswick, NJ, USA). When multi-axial images were obtained from one device, a vertical image was selected. When multi-source anterior segment OCT images of a patient were available, a study image was selected randomly using Python version 3.8. When data for both eyes of a patient were available, a study eye was selected randomly. In the training/validation and test datasets, the patients with no anterior segment OCT images of the crystalline lens, not obtained due to corneal opacity or other reasons, were excluded. All OCT images in the training/validation and test datasets were labeled with the preoperative BCVA (Figure 1).

### Artificial Intelligence Modeling

In this study, to improve the model performance and to analyze the technical error, we manipulated the AI modeling through reduction of input image size, use of image augmentation in the training dataset, and modification of the model architecture. The image size was reduced from 100 to 1% in 1% increments while maintaining the same height-to-width ratio. The multi-source OCT images varied in size, contour, and direction; the image augmentation method using ImageDataGenerator from Keras library was used for the training dataset. Finally, AI modeling was based on an ensemble of the Inception-v4 convolutional neural network (CNN) and ResNet via the stacking technique (23, 24). In the high-resolution and large-scale images, such as the OCT images in this study, a very deep CNN architecture can be expected to perform well until a certain level (25). However, exploding calculation and gradient vanishing are problems associated with very deep CNNs. To overcome these problems, architectures, such as the Inception network and ResNet, can be considered (23, 24). Ensemble modeling of the Inception network and ResNet was implemented using an aggregating method with a weighted average, and the hyperparameters of the model were modified to ensure that the model performed flexibly according to the proportion of the input image shape.

### Clinical Assessments

Clinical assessments were used to evaluate AI performance and perform error analysis in the medical approach. All patients underwent detailed preoperative examinations, including slit-lamp biomicroscopy, non-contact tonometry, ophthalmoscopy, and manifest refraction for BCVA. The mean corneal power and corneal astigmatism were measured using autokeratometry (Topcon KR-800A; Topcon Corporation, Tokyo, Japan). Intraocular lens calculation was performed using the IOLMaster® 700, and axial length and lens thickness were measured. An A-scan ultrasound biometry was used for intraocular lens calculation when the IOLMaster® 700 was unavailable. Cataract grading was performed using the Lens Opacities Classification System (LOCS) III by an expert surgeon (T.K.) who evaluated the opacity of the cortex, nucleus, and posterior subcapsular portion of the crystalline lens (26). Postoperative examinations, BCVA, slit-lamp examination, and pupil-dilation were conducted 1 month after the cataract surgery. Vision-impairing disease was defined as a clearly diagnosed disease in the detailed pre- and postoperative examinations that satisfied all of the following criteria: (1) postoperative BCVA > 0.1 logMAR, (2) persistent disease (that leaves an irreversible visual sequelae), (3) existing before cataract surgery, and (4) not a complication of cataract surgery.

### Model Performance

To evaluate the model performance, the mean absolute error (MAE), root mean square error (RMSE, which is influenced by large errors), and mean percentage error (MPE, which shows in percentage how much the forecasts of a model differ from the actual values) between the actual preoperative BCVA and predicted preoperative BCVA were calculated as follows:


$$MAE\;=\;\frac{\sum |\;y\;-\;\hat{y}\;|}{n}$$

$$RMSE=\;\sqrt{\frac{\sum {\left(y\;-\;\hat{y}\;\right)}^{2}}{n}}$$

$$\begin{array}{l} MPE\;=\;\frac{\sum (y\;-\;\hat{y}\;)}{n}\;\times 100\;(\%) \end{array}$$

y: true preoperative BCVA

ŷ: estimated preoperative BCVA

*n*: number of images in the test dataset

A correlation analysis was conducted between the true and estimated preoperative BCVAs with Pearson correlation coefficient.

### Error Analysis

The participants were initially classified into two groups based on the absolute error (AE) between true preoperative BCVA and estimated preoperative BCVA, one with an AE of at least 0.1 logMAR (AE_≥0.1_), and the other with AE under 0.1 logMAR (AE_ <0.1_). Next, the AE_≥0.1_ group was further divided into the underestimation group, in which the estimated preoperative BCVA was lower than the true preoperative BCVA, and the overestimation group, in which the estimated preoperative BCVA was higher than the true preoperative BCVA. Ordinal interference was considered if the clinical and statistical sequences were consistent.

### Statistical Analysis

Comparative analyses of clinical assessments were conducted between the groups and between the subgroups with independent *t-*tests for continuous variables and Fisher's exact tests for categorical variables. The values from the A-scan ultrasound biometry were excluded in the comparisons of axial length and lens thickness. Statistical significance was set at *p* < 0.05.

## Results

Table 1 shows the overall demographics and characteristics of the training/validation and test datasets. Although the OCT image source and underlying disease differed between the datasets, no remarkable differences were observed in age, sex, preoperative BCVA, axial length, lens thickness, and LOCS III cataract grade. The postoperative BCVA differed between the datasets (*p* < 0.001).

**Table 1:** Demographics and characteristics of the training/validation dataset and test dataset.

	**Training/validation dataset** **(*n* = 2,332)**	**Test dataset** **(*n* = 1,002)**
OCT image source	ANTERION® (*n* = 580) IOL-master® 700 (*n* = 1166) LenSx® laser system (*n* = 586)	IOL-master® 700 (*n* = 621) CATALYS™ laser system (*n* = 381)
Underlying disease	Senile cataract only	Senile cataract and/or other vision-impairing condition
Age (years, mean ± SD)	67.5 ± 9.1	69.1 ± 7.9
Sex (% of female)	64.5	63.9
Preoperative BCVA (logMAR, mean ± SD)	0.22 ± 0.15	0.20 ± 0.51
Postoperative BCVA (logMAR, mean ± SD)	0.00 ± 0.00	0.08 ± 0.12
Axial length (mm, mean ± SD)^*^	24.3 ± 1.2 (*n* = 2,186)	24.4 ± 1.3 (*n* = 948)
Lens thickness (mm, mean ± SD)^*^	4.3 ± 0.6 (*n* = 2,186)	4.3 ± 0.7 (*n* = 948)
**LOCS III cataract grade**		
Cortical (mean ± SD)	2.9 ± 1.3	3.0 ± 1.0
Nucleus (mean ± SD)	3.0 ± 0.6	3.0 ± 0.5
Posterior subcapsule (mean ± SD)	0.9 ± 1.5	1.0 ± 1.3

*BCVA, best corrected visual acuity; LOCS III, Lens Opacities Classification System III; logMAR, logarithm of the minimum angle of resolution; OCT, optical coherence tomography; SD, standard deviation*.

* *Considered the values of optical biometry*.

**Figure 2:**
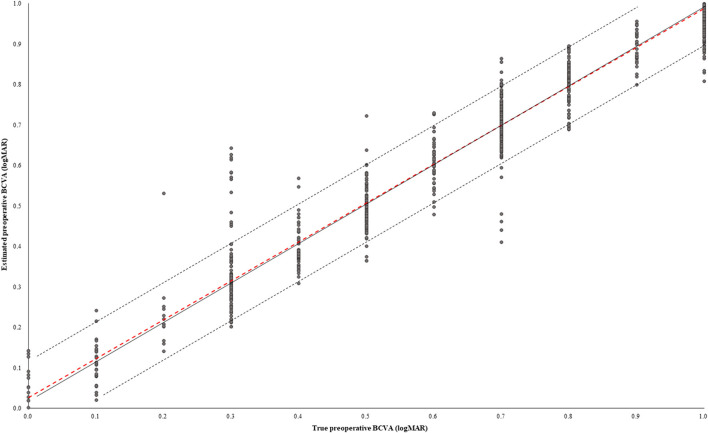
Scatter plot between true preoperative BCVA and estimated preoperative BCVA. The solid black line shows that the true and estimated preoperative BCVAs were equal. The black dotted line shows the absolute error (AE) of the true and estimated preoperative BCVAs of 0.1 logMAR. The red dotted line describes the Pearson correlation coefficient. In the test dataset, 91.6% of cases had AE under 0.1 logMAR.

In the test dataset, the MAE, RMSE, and MPE of the model performance were 0.050 ± 0.130 logMAR, 0.140 ± 0.134 logMAR, and 1.3 ± 13.9%, respectively (Table 2). The correlation coefficient (R) between the true preoperative BCVA and estimated preoperative BCVA was 0.969 (*p* < 0.001) (Figure 2). The percentage of cases in the AE ≥ 0.1 group was 8.4%, and 1.9% had AE ≥ 0.2.

**Table 2:** Performance of the artificial intelligence model for prediction of preoperative best corrected visual acuity in patients with senile cataract.

**Performance parameter**	**Value**
MAE (logMAR, mean ± SD)	0.050 ± 0.130
RMSE (logMAR, mean ± SD)	0.140 ± 0.134
MPE (%, mean ± SD)	1.3 ± 13.9
Correlation coefficient (*R*)	0.969 (*p* < 0.001)

*logMAR, logarithm of the minimum angle of resolution; MAE, mean absolute error; MPE, mean percentage error; RMSE, root mean square error; SD, standard deviation*.

The preoperative BCVA values were significantly different between the AE_≥0.1_ and AE_ <0.1_ groups (0.30 ± 0.08 vs. 0.12 ± 0.10, respectively; *p* < 0.001), and corneal astigmatism, lens thickness, and nucleus cataract in the LOCS III grading were significantly different (*p* < 0.001, < 0.001, and 0.024, respectively) (Table 3). Postoperative BCVA in the AE_≥0.1_ group was worse than that in the AE_ <0.1_ group (*p* < 0.001). In the AE_≥0.1_ group, the incidence of postoperative BCVA > 0.1 was 21.4%, which was higher than that in the AE_ <0.1_ group, and the proportion of vision-impairing disease was also higher than in the AE_ <0.1_ group. The percentage of cases with vision-impairing diseases was significantly higher in the AE_≥0.1_ group than in the AE_ <0.1_ group (54.8 vs. 12.9%; *p* < 0.001).

**Table 3:** Comparison of the patients showing absolute error of BCVA of 0.1 and over (AE_≥0.1_) with those showing absolute error under 0.1 (AE_<0.1_) in the test dataset.

	**AE_**≥0.1**_ (*n* = 84)**	**AE_**<0.1**_** **(*n* = 918)**	***p*** **-value**
OCT image source (% of CATALYS™ laser system)^‡^	35.7	35.9	0.966^*^
Mean age (years, mean ± *SD*)^§^	70.5 ± 8.2	69.0 ± 7.9	0.180
Sex (% of female)^‡^	64.3	63.8	0.934
Preoperative BCVA (logMAR, mean ± SD)^§^	0.30 ± 0.08	0.12 ± 0.10	<0.001^*^
Preoperative corneal power (D, mean ±*SD*)^§^	43.9 ± 1.6	43.9 ± 1.2	1.000
Preoperative corneal astigmatism (D, mean ± SD)^§^	1.2 ± 1.1	0.8 ± 0.5	<0.001^*^
Axial length (mm, mean ±*SD*)^†^^§^	24.6 ± 0.9 (*n* = 75)	24.4 ± 1.4 (*n* = 873)	0.225
Lens thickness (mm, mean ± SD)^†^^§^	4.6 ± 0.2 (*n* = 75)	4.3 ± 0.7 (*n* = 873)	<0.001^*^
**LOCS III cataract grade**			
Cortical (mean ± SD)^§^	2.9 ± 1.1	3.0 ± 1.0	0.436
Nucleus (mean ± SD)^§^	3.1 ± 0.3	3.0 ± 0.3	0.024^*^
Posterior subcapsule (mean ± SD)^§^	0.9 ± 1.6	1.0 ± 1.3	0.491
Postoperative BCVA (logMAR, mean ± SD)^§^	0.07 ± 0.05	0.01 ± 0.09	<0.001^*^
Postoperative BCVA > 0.1 (%)^‡^	21.4	6.8	<0.001^*^
**Vision-impairing disease (%)** ^‡^	54.8	12.9	<0.001^*^
Corneal disease, central (%)^‡^	4.8	0.0	<0.001^*^
Macular disease (%)^‡^	26.2	7.2	<0.001^*^
Glaucoma and other optic neuropathy (%)^‡^	28.6	6.3	<0.001^*^

* *p-value < 0.05*.

† *Considered the values of optical biometry only*.

‡ *Analyzed by Fisher's exact test*.

§ *Analyzed by independent t-test*.

*BCVA, best corrected visual acuity; D, diopter; LOCS III, Lens Opacities Classification System III; logMAR, logarithm of the minimum angle of resolution; SD, standard deviation*.

The AE_≥0.1_ group was further divided into underestimation and overestimation groups (Table 4). Pre- and postoperative BCVAs were significantly worse in the underestimation group (0.39 ± 0.10 *vs*. 0.15 ± 0.09 in overestimation, and 0.10 ± 0.15 vs. 0.01 ± 0.05; *p* < 0.001 and *p* < 0.001, respectively). The proportion of cases with postoperative BCVA > 0.1 was higher in the underestimation group (*p* < 0.001). The proportion of cases with vision-impairing disease was 95.7% in the underestimation group. Axial length was longer, and cortical opacity and posterior subcapsular opacity were more severe in the underestimation group (*p* < 0.001, 0.045, and 0.002, respectively).

**Table 4:** Comparison of the overestimation and underestimation groups among patients showing an absolute error of 0.1 and over (AE_≥0.1_) in BCVA.

	**Underestimation (*n* = 46)**	**Overestimation** **(*n* = 38)**	***p*** **-value**
OCT image source (% of CATALYS™ laser system)^‡^	37.0	34.0	0.794
Mean age (years, mean ± SD)^§^	69.6 ± 7.5	71.8 ± 8.2	0.203
Sex (% of females)^‡^	65.2	63.2	1.000
Preoperative BCVA (logMAR, mean ± SD)^§^	0.39 ± 0.10	0.15 ± 0.09	<0.001^*^
Preoperative corneal power (D, mean ± SD)^§^	43.8 ± 1.4	44.2 ± 1.7	0.240
Preoperative corneal astigmatism (D, mean ± SD)^§^	1.2 ± 1.1	1.1 ± 1.0	0.677
Axial length (mm, mean ± SD)^†^	25.1 ± 1.0 (*n* = 37)	24.2 ± 0.8	<0.001^*^
Lens thickness (mm, mean ± SD)^†^	4.5 ± 0.2 (*n* = 37)	4.6 ± 0.2	<0.034^*^
**LOCS III cataract grade**
Cortical (mean ± SD)^§^	3.3 ± 1.0	2.6 ± 1.0	>0.045^*^
Nucleus (mean ± SD)^§^	3.0 ± 0.5	3.1 ± 0.2	0.775
Posterior subcapsule (mean ± SD)^§^	1.5 ± 1.6	0.3 ± 0.6	>0.002^*^
Postoperative BCVA (logMAR, mean ± SD)^§^	0.10 ± 0.15	0.01 ± 0.05	<0.001^*^
Postoperative BCVA >0.1 [% of each group, (% of AE_≥0.1_)]^‡^	34.8 (88.9)	5.3 (11.1)	<0.001^*^
Vision-impairing disease [% of each group, (% of AE_≥0.1_)]^‡^	95.7 (95.7)	5.3 (4.3)	<0.001^*^

* *p-value < 0.05*.

† *Considered the values of optical biometry only*.

‡ *Analyzed by Fisher's exact test*.

§ *Analyzed by independent t-test*.

*BCVA, best corrected visual acuity; D, diopter; LOCS III, Lens Opacities Classification System III; SD, standard deviation*.

Error analysis for model performance was conducted by performing comparative analyses between the AE_≥0.1_ and AE_ <0.1_ groups and between the underestimation and overestimation subgroups of the AE_≥0.1_ group (Table 5). Preoperative corneal astigmatism, lens thickness, and nuclear opacity in the LOCS III grading were higher in the AE_≥0.1_ group. Increased BCVA and vision-impairing disease were more significant in the underestimation subgroup. Axial length was longer, and the cortical/posterior subcapsular opacity was more severe in the overestimation subgroup than in the underestimation subgroup.

**Table 5:** Error analysis of model performance in clinical practice.

**Characteristics**	**AE_**≥0.1]**_ vs. AE_**<0.1**_**	**Estimation group**		
		**Under**	**AE_**<0.1**_ (fair)**	**Over**
OCT image source	–	–	–	–
Age	–	–	–	–
Sex	–	–	–	–
Preoperative BCVA	+	+	–	–
Preoperative corneal astigmatism	+	+	–	+
Postoperative BCVA	+	+	–	–
Postoperative BCVA over 0.1	+	+	–	–
Vision-impairing disease	+	+	–	–
Axial length^†^	–	+^longer^	+^intermediate^	+^shorter^
Lens thickness^‡^	+	+	–	+
**LOCS III grade**				
Cortex^†^	–	+^higher^	+^intermediate^	+^lower^
Nucleus	+	+	–	+
Posterior subcapsule^†^	–	+^higher^	+^intermediate^	+^lower^

† *Ordinal interference*.

‡ *Statistically different between the underestimation and overestimation groups but failed to list in order of lens thickness*.

**Figure 3:**
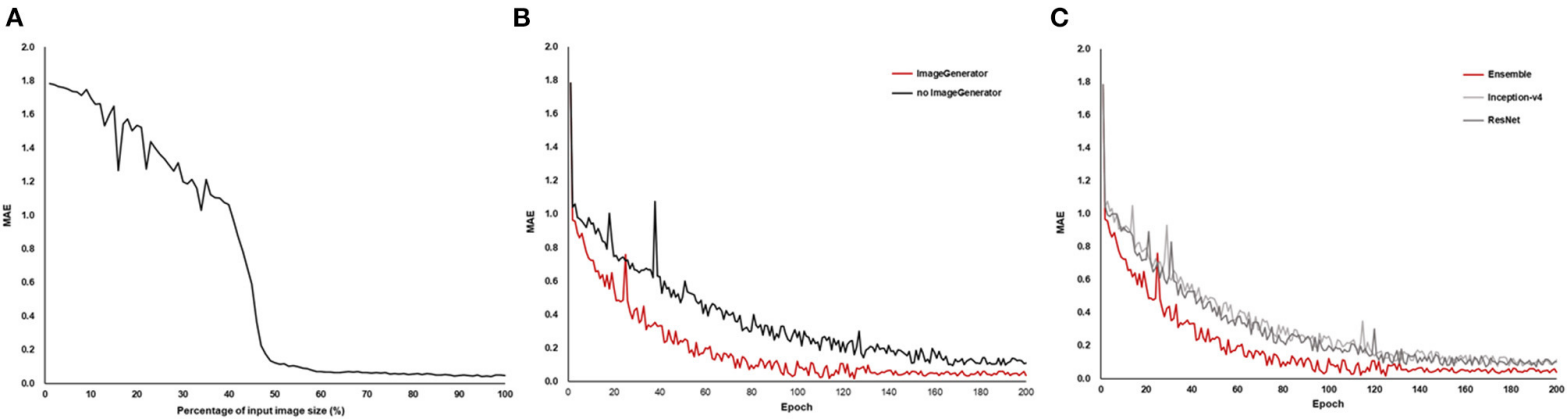
Mean absolute error (MAE) of the model performance in the AI training process. As the size of the OCT image reduced, MAE increased **(A)**, and image augmentation using the image generator showed a lower MAE **(B)**. The ensemble model of Inception-v4 and ResNet showed the lowest MAE compared to the individual architecture **(C)**.

In the AI modeling process, reducing the original image decreased the model performance, and the neural network could not estimate the BCVA when the area was reduced by ~≥90% (32% of width and height) (Figure 3A). Image augmentation increased the model performance in terms of the MAE (Figure 3B). The ensemble model had the lowest MAE, and Inception-v4 and ResNet were almost similar in terms of MAE (Figure 3C).

## Discussion

With the test dataset of this study, over 90% of cases could be estimated in their BCVA under 0.1 logMAR of AE. Most of the underestimation errors were caused by vision-impairing disease, and about half of the decreased model performance could be explained by a clinical approach. The technical issues, image resolution, diversity of image forms in the training dataset, and model architecture also affected the model performance.

Objective clinical assessment for estimation of the subjective visual symptoms represented by visual acuity, which is considered the ultimate goal of ophthalmologic interventions, can be utilized in various ways from clinical to experimental; however, this is very difficult for clinicians due to confounding factors (27). AI is expected to help human evaluators perform difficult tasks (28). This study suggested that cataract severity can be quantified as visual acuity via AI using OCT images.

In this study, cross-instrument validation was performed using different combinations of anterior segment OCT image sources for the training/validation dataset and the test dataset. To enhance the model performance, the OCT images used in the training/validation dataset were obtained from three devices with different detection methods, image resolutions, and image directions. The test dataset was constructed to evaluate the practical application of the AI model in real-world scenarios by including all patients within a certain period and using commonly available OCT images in the process of cataract surgery and its preparation.

Model performance and error analysis were conducted from both clinical and engineering perspectives. Although medical issues have rarely been mentioned in previous AI research, for clinical application of medical AI, error analysis from the clinical perspective is indispensable (29, 30). The results suggest that the predisposing disease and the conditions, such as high values of corneal astigmatism, axial length, lens thickness, and severity of each cataract subtype, which are well known to cause visual impairment, were the main factors underlying errors in the medical approach (8, 31–34). For engineering issues, this study suggested that low-resolution images led to the degradation of the model performance (see Figure 3A), and highlighted the importance of high-resolution images in analyzing precise medical observations (35). Multi-source images were used, and the transformation for direction and size through image augmentation in the training dataset was shown to yield better model performance (36). Although the AI model using a unified material may show a different performance from that using multi-sources materials, we focused on the versatility of the AI model by using OCT images acquired with multiple instruments.

The primary goal of cataract surgery is to restore the best possible vision as well as remove the natural crystalline lens (37). Thus, postoperative visual acuity is the target indicator of cataract surgery. Wei et al. reported the use of AI with fundus OCT for predicting postoperative visual acuity in patients with high myopia (38), and the result showed the lowest MAE of 0.16 logMAR and RMSE of 0.24 logMAR. However, this performance level was still insufficient for clinical application, and the findings implied that visual acuity was not determined by a single defined etiology. Clinicians frequently encounter patients with visual impairment without a specific pathologic lesion. This phenomenon may be caused by medical issues and unknown predisposing factors, including developmental problems, such as amblyopia, an extraocular disease, such as brain lesion, or a temporary problem, such as dry eye disease. Thus, approaches based on a single etiological factor showed limited ability to predict the postoperative BCVA. Since multiple factors, including the presence of both cataract and comorbid disease, can influence the accuracy of prediction of postoperative BCVA with AI based on the preoperative BCVA, our study could serve as the basis for future studies. Future studies should aim to predict postoperative visual acuity using a combination of preoperative visual acuity, anterior segment OCT, and other examinations.

**Figure 4:**
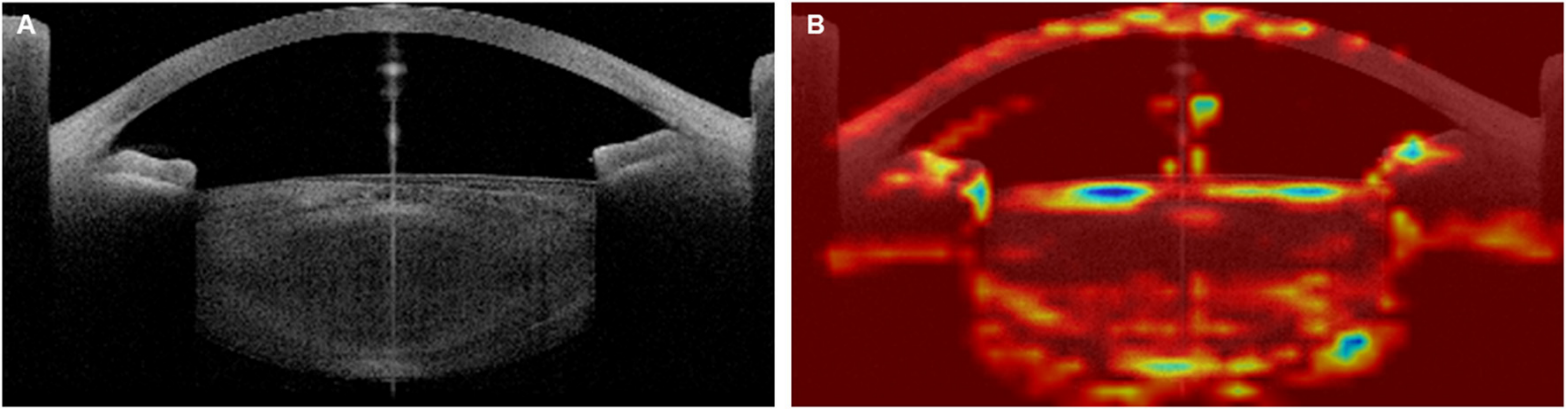
Example of heatmap analysis of the Artificial Intelligence (AI) attention area. Original OCT image **(A)** and Heatmap image **(B)**. The preoperative best-corrected visual acuity (BCVA) of this 67-year-old patient, who showed a senile cataract with grade 3 cortical opacity and grade 2 nuclear opacity in the Lens Opacities Classification System III, was 0.50 logMAR, and the AI-estimated BCVA was 0.52 logMAR.

Similar to previous medical AI research (16, 39–41), this study did not completely overcome the limitations in interpreting the neural network. In this study, the attention areas of the AI model were analyzed using heatmap analysis (Figure 4), and we tried to analyze the relationships between the attention areas and the error analysis in the medical approach. Although model training was conducted using patients with only a clinical diagnosis of cataract, the AI also recognized the cornea that was laid on the visual axis and the angle in the anterior chamber. Thus, some factors affecting the corneal shape (e.g., corneal refractive surgery), anterior chamber angle (e.g., glaucoma), and lens thickness may be related.

The strengths of this study were not limited to the excellent performance of the AI model. Our attempt to prove the importance of clinical approach in model performance was successful and our findings suggest that the addition of various clinical information in AI modeling is crucial for improving model performance.

In conclusion, the AI developed using OCT images from multiple sources showed excellent performance in estimating visual acuity in patients with senile cataracts. This quantification method encompasses both visual acuity and cataract severity of the OCT images, which are the main indications for cataract surgery, and has the potential to allow objective evaluation of cataract severity. This AI model can be used when it is difficult to express or measure the subjective visual acuity due to various causes, such as an inability to communicate. Additionally, we would like to emphasize that this was a preliminary study to expand the prediction of visual acuity after cataract surgery in patients with other diseases, possibly accompanied by visual impairment.

## Data Availability Statement

The datasets presented in this article are not readily available because of privacy and ethical concerns, neither the data nor the source of the data can be made available. Requests to access the datasets should be directed to HA, overhyun31@gmail.com.

## Ethics Statement

The studies involving human participants were reviewed and approved by the Severance Hospital Clinical Research Ethics Committee. Written informed consent for participation was not required for this study in accordance with the national legislation and the institutional requirements.

## Author Contributions

HA and T-iK: conceptualization. HA: methodology, software, formal analysis, investigation, writing—original draft preparation, and visualization. IJ, KS, EK, and T-iK: validation. IJ, KS, and T-iK: resources. T-iK: writing—review and editing and supervision. All authors contributed to the article and approved the submitted version.

## Conflict of Interest

The authors declare that the research was conducted in the absence of any commercial or financial relationships that could be construed as a potential conflict of interest.

## Publisher's Note

All claims expressed in this article are solely those of the authors and do not necessarily represent those of their affiliated organizations, or those of the publisher, the editors and the reviewers. Any product that may be evaluated in this article, or claim that may be made by its manufacturer, is not guaranteed or endorsed by the publisher.

## Abbreviations

AE: absolute error
AI: artificial intelligence
BCVA: best-corrected visual acuity
CNN: convolutional neural network
LOCS: Lens Opacities Classification System
MAE: mean absolute error
MPE: mean percentage error
OCT: optical coherence tomography
RMSE: root mean square error.
